# Development of an *in vitro* potency assay for human skeletal muscle derived cells

**DOI:** 10.1371/journal.pone.0194561

**Published:** 2018-03-22

**Authors:** Marco Thurner, Faheem Asim, Dorota Garczarczyk-Asim, Katrin Janke, Martin Deutsch, Eva Margreiter, Jakob Troppmair, Rainer Marksteiner

**Affiliations:** 1 Innovacell Biotechnologie AG, Science Park, Innsbruck, Austria; 2 Daniel Swarovski Research Laboratory, Department of Visceral, Transplant, and Thoracic Surgery, Medical University of Innsbruck, Innsbruck, Austria; Murdoch Childrens Research Institute, AUSTRALIA

## Abstract

**Background:**

Potency is a quantitative measure of the desired biological function of an advanced therapy medicinal product (ATMP) and is a prerequisite for market approval application (MAA). To assess the potency of human skeletal muscle-derived cells (SMDCs), which are currently investigated in clinical trials for the regeneration of skeletal muscle defects, we evaluated acetylcholinesterase (AChE), which is expressed in skeletal muscle and nervous tissue of all mammals.

**Methods:**

CD56^+^ SMDCs were separated from CD56^-^ SMDCs by magnetic activated cell sorting (MACS) and both differentiated in skeletal muscle differentiation medium. AChE activity of *in vitro* differentiated SMDCs was correlated with CD56 expression, fusion index, cell number, cell doubling numbers, differentiation markers and compared to the clinical efficacy in patients treated with SMDCs against fecal incontinence.

**Results:**

CD56^-^ SMDCs did not form multinucleated myotubes and remained low in AChE activity during differentiation. CD56^+^ SMDCs generated myotubes and increased in AChE activity during differentiation. AChE activity was found to accurately reflect the number of CD56^+^ SMDCs in culture, their fusion competence, and cell doubling number. In patients with fecal incontinence responding to SMDCs treatment, the improvement of clinical symptoms was positively linked with the AChE activity of the SMDCs injected.

**Discussion:**

AChE activity was found to truly reflect the *in vitro* differentiation status of SMDCs and to be superior to the mere use of surface markers as it reflects not only the number of myogenic SMDCs in culture but also their fusion competence and population doubling number, thus combining cell quality and quantification of the expected mode of action (MoA) of SMDCs. Moreover, the successful *in vitro* validation of the assay proves its suitability for routine use. Most convincingly, our results demonstrate a link between clinical efficacy and the AChE activity of the SMDCs preparations used for the treatment of fecal incontinence. Thus, we recommend using AChE activity of *in vitro* differentiated SMDCs as a potency measure in end stage (phase III) clinical trials using SMDCs for skeletal muscle regeneration and subsequent market approval application (MAA).

## Introduction

Personalized cell-based therapies have opened new possibilities to treat previously incurable diseases and have significantly improved the quality of life for many patients [1]. The need to provide safe, stable and fully evaluated products is becoming an important task for developers, manufacturers and regulators. Potency evaluation of a cell-based therapy is an integral part in the assessment of overall quality, along with parameters such as viability, purity, efficacy and dose (number of cells). From a European regulatory perspective, potency is defined as a quantitative measure of the desired biological function of an advanced therapy medicinal product (ATMP) and is a prerequisite for a market approval application (MAA) under European Commission directive 2009/120/EC (EMA Directives, 2009) [2]. Potency has a central role in an ATMP development, providing a link between quality attributes and clinical efficacy that ultimately leads to a dose definition. Ideal *in vitro* candidates for a potency assay include a specific mRNA, peptide, enzyme, small molecule, growth factor, cytokine or receptor etc., which is quantifiable and represents the desired mode of action (MoA) of a cell therapy product. The potency assay accounts for key process- and product-related parameters (stability and quality) and is measureable at every step during the process. In the clinical development of ICEF15, a skeletal muscle-derived cells (SMDCs) based ATMP aiming the regeneration of skeletal muscle tissue of the *M*. *sphincter ani externus*, the availability of a potency assay for continuation of our clinical program (currently ongoing Phase IIb trial; EudraCT: 2010-021463-32) towards end stage clinical trials has become mandatory. To this end our work focused on acetylcholinesterase (EC 3.1.1.7; AChE), which belongs to the type B carboxylesterases and is found in skeletal muscle, nervous tissue and erythrocytes of all mammals [3]. AChE is primarily active in the cholinergic synapses and neuromuscular junctions [3–6]. The principal function of AChE is to regulate the nerve impulse transmission by hydrolysis of the neurotransmitter acetylcholine [7]. AChE is a polymorphic enzyme and from its gene *ACHE*, many different catalytic subunits are produced by alternative splicing [8,9]. Only the type T AChE splice variant (AChE_T_) is expressed in mammalian muscle and brain [8,9]. Ellman’s method allows AChE quantification in a variety of biological samples ranging from whole blood or serum to different animal tissues and cell lines [10].

In the body, skeletal muscle tissue is regenerated upon injury by activation of quiescent satellite cells that give rise to proliferating myoblasts that are able to fuse with each other and the existing fibers by cell differentiation in order to restore muscle function [11–13]. Cassar-Malek et al. observed that myoblasts started to form myotubes after 3 days in differentiation medium by cell to cell fusion. The AChE activity was low in proliferating myoblasts but it increased sharply during cell to cell fusion [14]. Siow et al. reported a 5-fold increase in AChE activity in differentiated mouse myoblasts over 6 days [15]. The expression and distribution of AChE in human myotubes were studied by Mis et al. in an *in vitro* model of innervated human muscle by co-culturing rat embryonic spinal cord explant with human myotubes showing that AChE is expressed by muscle cells and neurons [16]. In a similar investigation of an *in vitro* model of innervated human muscle and rat embryonic spinal cord explant, Jevsek et al. reported a significant muscular AChE contribution at the neuromuscular junction (NMJ) [17], suggesting that the increase in muscle AChE activity during differentiation may be relevant for physiological functionality of mature NMJs.

Measurement of a parameter that represents the MoA and potency of SMDCs would allow implementing a cut off value, which has to be reached for the release of *in vitro* preparations of SMDCs for their clinical use. Mitterberger et al. isolated SMDCs from a small human muscle biopsy (about 0.3 cm^3^) [18,19]. These SMDCs were myogenic, as defined by the expression of CD56 and desmin, both considered to be myoblast markers [20–23], and underwent *in vitro* differentiation to multinucleated myotubes [18]. Myoblasts are the main myogenic cells observed in SMDCs, which originate from quiescent muscle satellite cells [24,25]. These SMDCs have been successfully used in clinical trials of fecal incontinence aiming, the regeneration of weakened external anal sphincter muscle [26,27].

In this work, we aimed to test whether measuring the AChE activity of *in vitro* differentiated human SMDCs can serve as a potency assay for SMDCs aiding functional muscle regeneration.

## Results

### AChE activity is a quantitative marker of SMDCs differentiation

**Fig 1:**
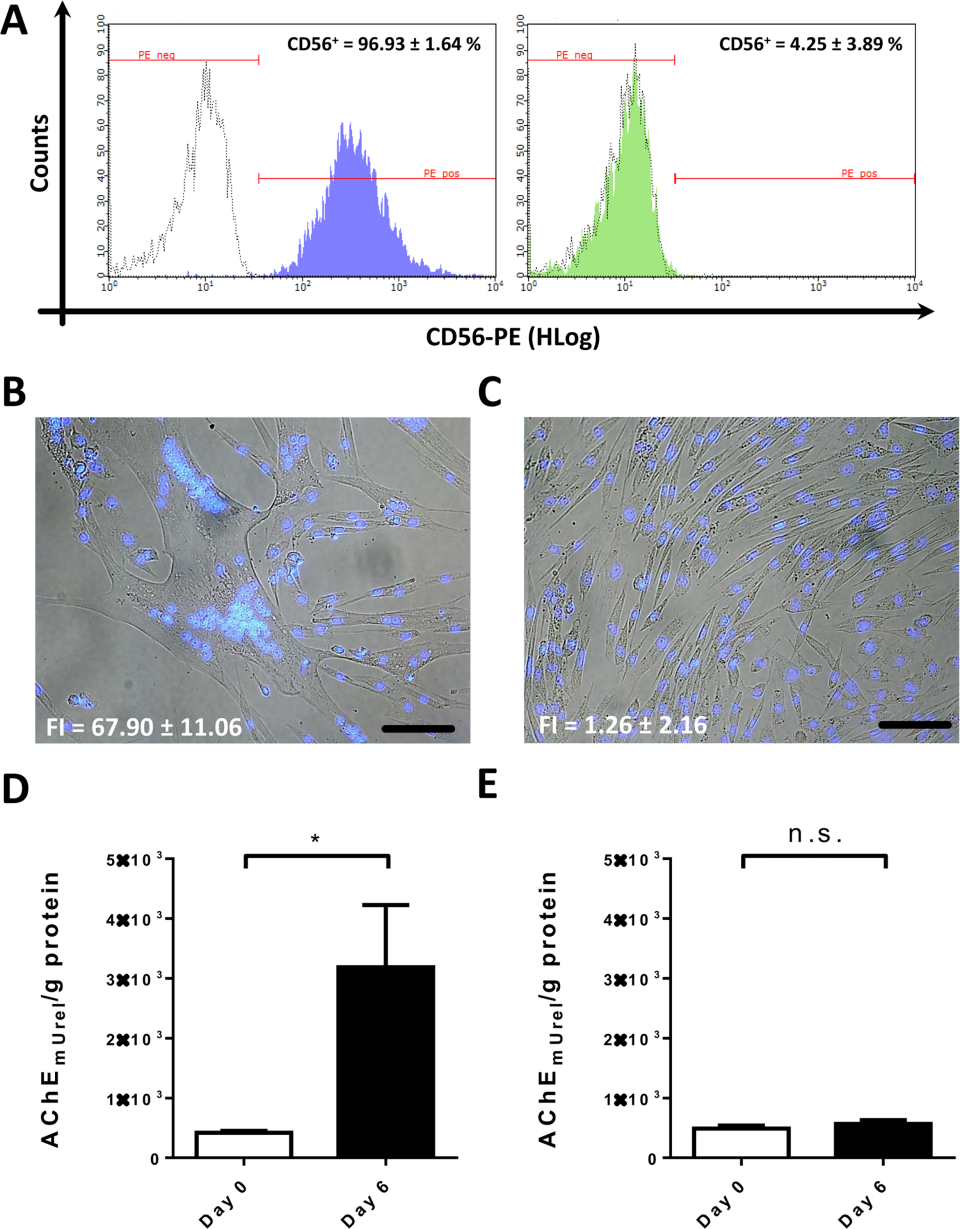
SMDC isolation, differentiation and AChE quantification. CD56^+^ and CD56^-^ cells were separated by MACS. Successful separation was confirmed by flow cytometry. Positively selected cells from five different human muscle biopsies using anti CD56 MACS antibody contain 96.93 ± 1.64% CD56^+^ cells (representative histogram in purple) whereas negatively selected cells from five individual patients contain 4.25 ± 3.89% CD56^+^ cells (representative histogram in green). White histograms represent isotype control staining (A). When cultured in skeletal muscle differentiation medium, single nucleated CD56^+^ SMDCs fuse and produce multinucleated tubes on gelatin coated 24-well plates with a fusion index (FI) of 67.90 ± 11.06 (mean ± SD; n = 3) after 6 days of differentiation (B). CD56^-^ SMDCs do not form myotubes within 6 days of cultivation in differentiation medium on gelatin coated 24-well plates (FI = 1.26 ± 2.16, n = 3) (C). The change in AChE activity per g total protein of 200 000 CD56^+^ cells seeded on gelatin-coated 24-well plates before induction of differentiation (Day 0) and 6 days after differentiation (Day 6) is significantly different (p<0.05) in a ratio paired *t*-test of at least three patient samples (data presented as mean ± SEM) (D) but not significantly (n.s.) different between AChE activity in three batches of CD56^-^ cells before and 6 days after induction of skeletal muscle differentiation in a ratio paired *t*-test of three different SMDC patient samples (E). Scale bar (white) = 100 μm.

The progression of human myoblast growth and fusion was observed in 24-well culture plates during the cultivation of CD56^+^ SMDCs (>95% CD56^+^), that had been separated from CD56^-^ SMDCs (<5% CD56^+^) *via* MACS (Fig 1A). CD56 is a myogenic marker whose expression directly correlates with desmin (S1 Fig). Cell differentiation was induced by switching from growth to skeletal muscle differentiation medium. Successful induction of myotube formation in CD56^+^ SMDCs was observed by light microscopy and staining of nuclei with Hoechst 33244 allowing for the calculation of the fusion index (FI) (Fig 1B). Myotubes reached maximum length and number of nuclei on day 6 (Fig 1B). This differentiation went along with a significant increase in AChE activity when comparing day 0 to day 6 (Fig 1D). In contrast, CD56^-^ SMDCs had not fused to multinucleated myotubes after 6 days in differentiation medium (Fig 1C) and did not significantly increase their AChE activity (Fig 1E). Based on these results we hypothesized, that the AChE activity during differentiation correlates with the percent of CD56^+^ SMDCs in a population as well with the fusion competence of the population.

**Fig 2:**
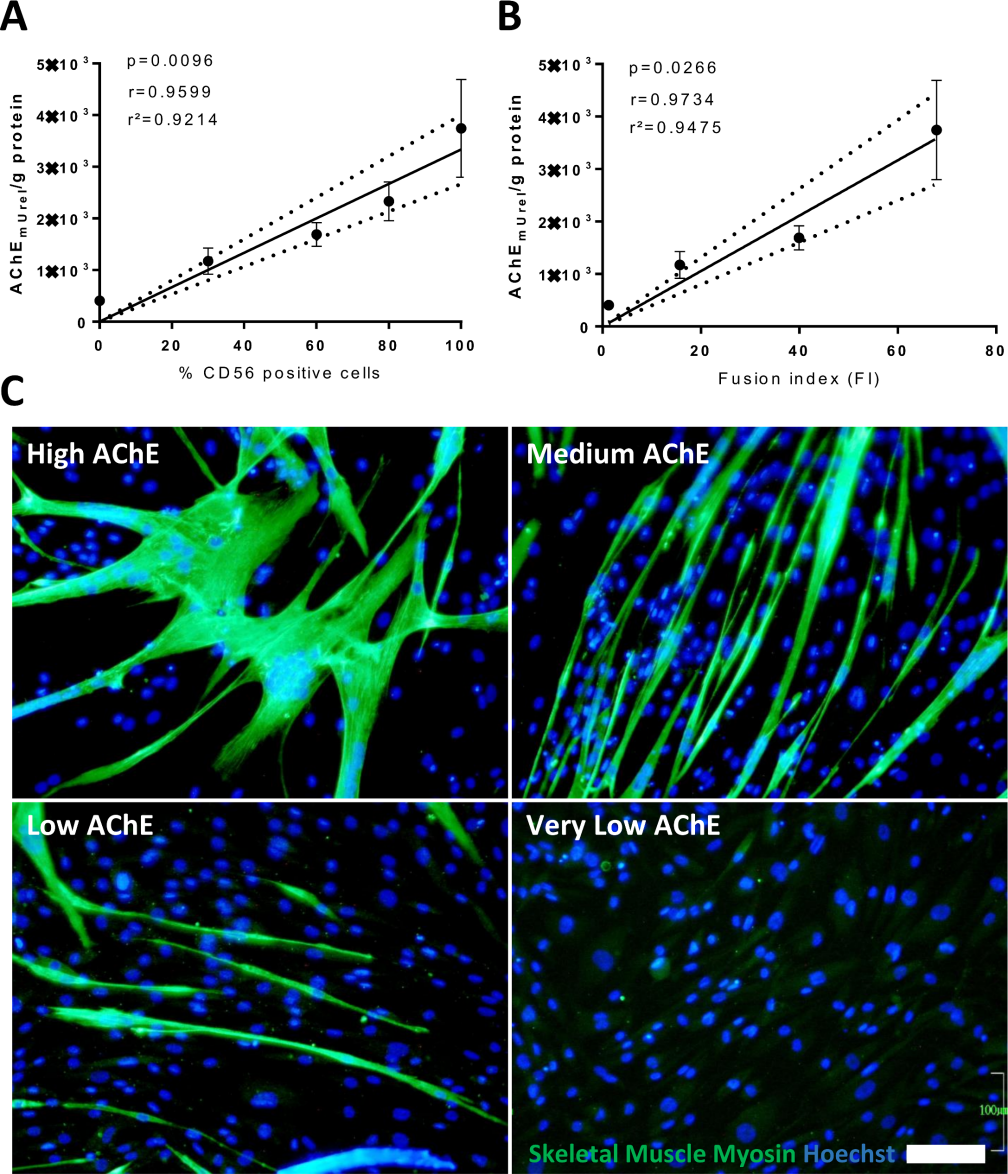
Comparison of AChE activity with CD56 expression and fusion competence. AChE activity per g of total protein was determined in SMDC populations derived from at least three different patients with different percentages of CD56^+^ cells after 6 days of differentiation on gelatin coated 24-well plates. Correlation between AChE and CD56 expression in SMDCs by Pearson linear regression analysis of at least 4 different patient samples results at a *p*-value of *p* = 0.0096, a correlation coefficient of *r* = 0.9599 and coefficient of determination of *r*^*2*^ = 0.9214. Data points were presented as mean±SEM AChE activity. 95% confidence intervals are shown as dotted lines (A). Correlation between AChE and fusion index (FI) of SMDCs by Pearson linear regression analysis of at least 4 different patient samples resulted in a *p*-value of *p* = 0.0266, a correlation coefficient of *r* = 0.9734 and coefficient of determination of *r*^*2*^ = 0.9475. Data points presented as mean±SEM AChE activity and mean±SEM fusion index of four different patient samples in a total of 16 mixtures of fusion competent and non-fusogenic cells. 95% confidence intervals are shown as dotted lines (B). SMDCs from 4 patients resulting in a total of 16 SMDCs populations grouped in “High AChE”, “Medium AChE”, “Low AChE” and “Very low AChE” according to their mean±SEM AChE activity of 4421±540, 1774±176, 1302±179 and 398±27 AChEmUrel/g protein, respectively stained for SK-Myosin expression after 6 days of differentiation on gelatin-coated 24-well plates. Scale bar = 100 μm (C).

To test the correlation between the AChE activity and the number of CD56^+^ SMDCs within the culture we measured the AChE activity of cultures containing various proportions of CD56^+^ and CD56^-^ cells (>95%, 80%, 60%, 30% and <5% CD56^+^ cells) after 6 days of differentiation. Linear regression analysis (Pearson) showed a highly significant correlation (*r* = 0.9599; *p* = 0.0096, *r*^*2*^ = 0.9214) (Fig 2A), suggesting that the AChE activity of the whole cell population is solely contributed by CD56^+^ SMDCs.

Next, we tested whether the fusion competence of SMDCs also correlates with their AChE activity. Various proportions of fusion competent (CD56^+^) and non-fusogenic cells (CD56^-^) were prepared and subsequently the AChE activity and the fusion index were calculated after 6 days of skeletal muscle differentiation. SMDCs populations derived from 4 independent muscle biopsies were analysed and mean fusion index was calculated and correlated with AChE activity. Linear regression analysis (Pearson) showed a highly significant correlation (*r* = 0.9734; *p* = 0.0266, *r*^*2*^ = 0.9475) (Fig 2B), suggesting that AChE activity reflects the fusion competence of the SMDCs.

Besides fusion competence, also the expression of differentiation marker for mature skeletal muscle is necessary to confirm effective skeletal muscle maturation of SMDCs. To test if AChE also correlates with maturation, populations of SMDCs with diverse AChE activity were grouped in “High AChE”, “Medium AChE”, “Low AChE” and “Very Low AChE” according to their mean±SEM AChE activity of 4421±540, 1774±176, 1302±179 and 398±27 AChE_mUrel_/g protein under differentiation conditions, respectively. These populations were cultured in skeletal muscle differentiation medium for 6 days and analysed for expression of the late stage skeletal muscle marker skeletal muscle (SK) myosin. Immunofluorescence images of representative populations shown in Fig 2C, demonstrate that no mature SK-myosin expressing myotubes were found in populations with very low AChE activity compared to an increasing number and size of SK-myosin positive myotubes in the respective SMDC populations (medium, high, and very high AChE activity). These results suggest that AChE activity directly correlates with the number and size of mature SK-myosin expressing myotubes upon skeletal muscle differentiation.

Comparison of *ACHE* gene expression of SMDCs before (day 0) and 6 days after induction of skeletal muscle differentiation by gene chip analysis (Affymetrix) revealed that the *ACHE* expression of SMDCs was upregulated when cultured in differentiation medium (Figure C in S2 Fig). Since total protein content and the total number of nuclei did not change during 6 days of differentiation (Figures A and B in S2 Fig), further analysis of AChE could be simplified by omitting total protein determination of SMDC batches manufactured during clinical trials.

**Fig 3:**
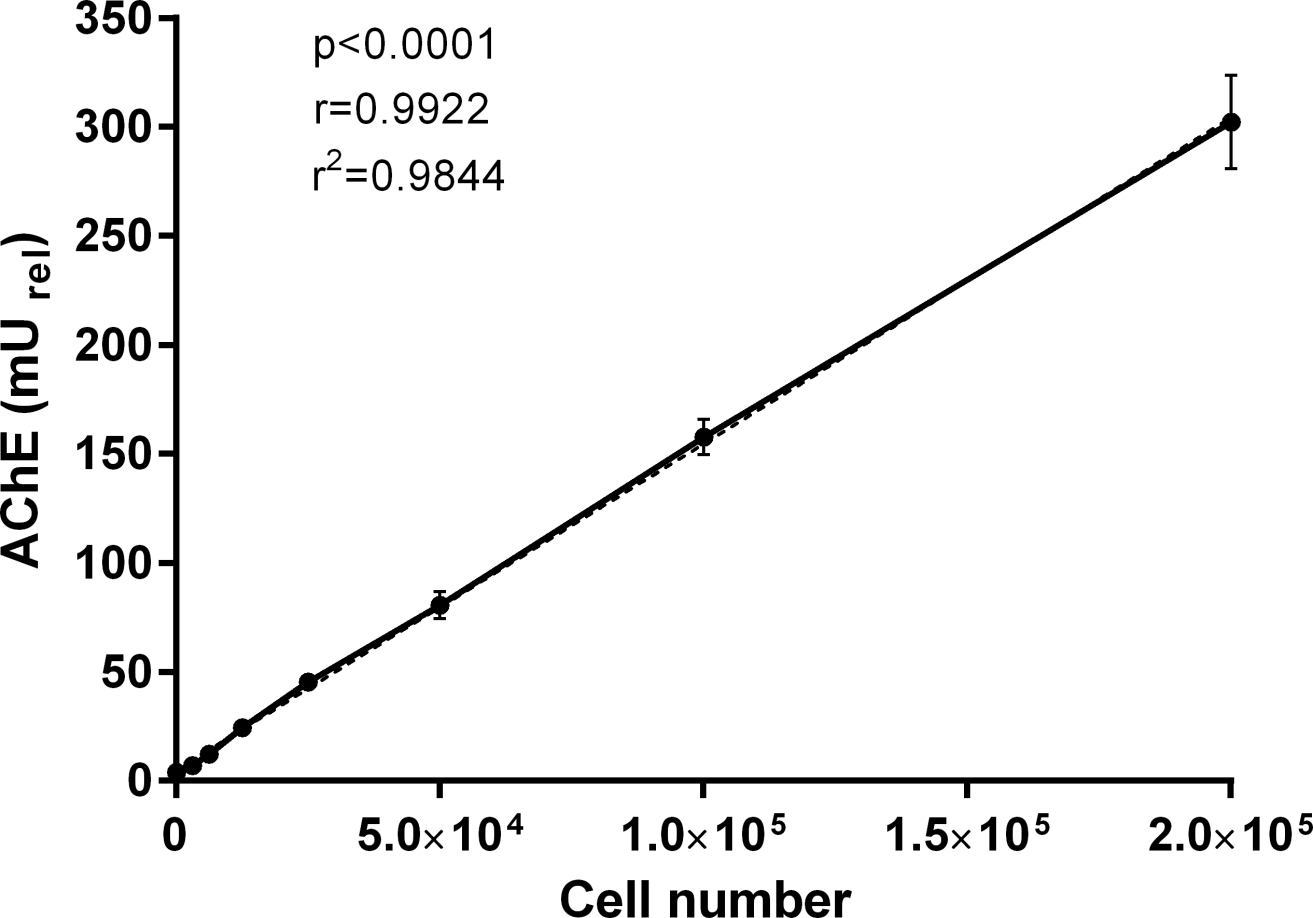
Correlation of AChE activity with cell number. Data presented as mean ± SEM of three individual experiments. Coefficient of determination (*r*^*2*^), correlation coefficient (*r*) and *p-value* (*p*) was calculated by Pearson linear regression analysis.

As SMDCs are a cell-based drug and the MoA is depending on single cells as active entities within the product we tested whether the AChE activity measured during SMDCs differentiation is cell number dependent. Therefore, we seeded different numbers (2.5*10^4^, 5*10^4^, 1*10^5^ and 2*10^5^) of CD56^+^ SMDCs onto gelatin-coated 24-well plates and induced them to differentiate. As shown in Fig 3, AChE activity strongly correlates well with the cell number (*r* = 0.9922, *p*<0.0001, *r*^*2*^ = 0.9844), suggesting that that AChE activity measured during SMDCs differentiation is dependent on number of cells seeded for differentiation.

### Effects of population doublings on AChE and SMDCs differentiation

**Fig 4:**
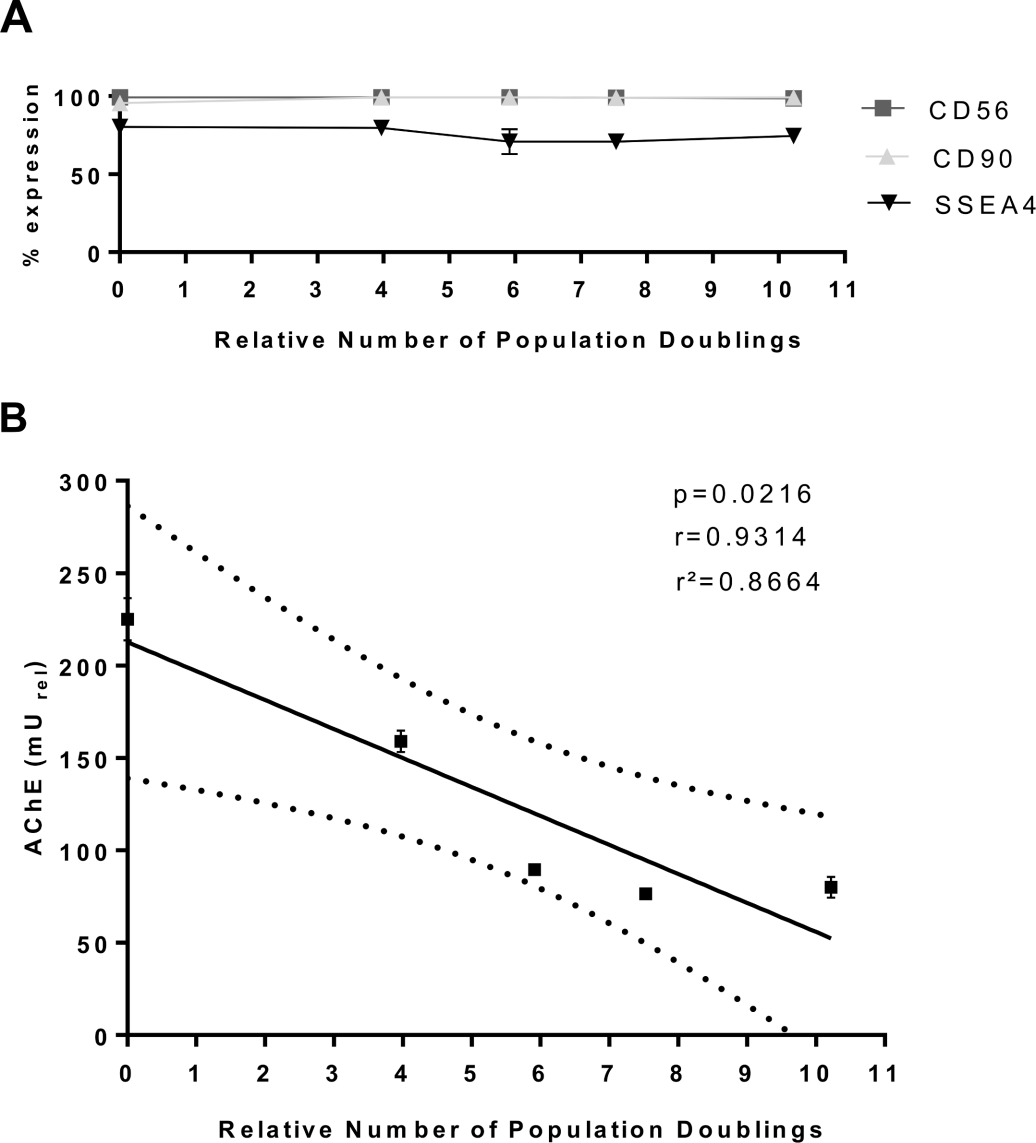
Cell doubling and AChE activity. Effect of cell doubling numbers on cell surface marker expression (CD56, CD90, SSEA-4) (A). AChE activity measured 6 days after differentiation of cells harvested after a total culture time of 6 days with differing numbers of medium changes during the cultivation period leading to different growth kinetics was correlated with the relative number of population doublings over 6 days of culture. Pearson linear regression analysis led to a *p*-value of *p* = 0.0216, a correlation coefficient of *r* = 0.9314 and a coefficient of determination of *r*^*2*^ = 0.8664. 95% confidence intervals are shown as dotted line (B). Data presented as mean±SEM of three individual experiments.

To understand whether AChE activity is a sensitive marker for the expansion of SMDCs in culture and whether it reflects a decrease in differentiation competency upon increased cell doubling, AChE activity and fusion index were calculated for SMDCs that had undergone different population doublings. For this, 4.25*10^6^ CD56^+^ SMDCs, which were controlled for initial AChE activity after 6 days of differentiation, and then seeded in parallel in 175 cm^2^ flasks in growth medium and cultivated for 6 days according to the following protocols: no medium change for 6 days, medium change on the third, every second or every day. Afterwards cells were harvested, the number of population doublings calculated, and the cells analysed by flow cytometry for the expression of CD56 and the stem cell specific markers CD90 and SSEA4. In the next step, these differently cultured cells were seeded for *in vitro* differentiation to assess their AChE activity and fusion index. As shown in Fig 4A neither CD56 nor SSEA-4 and CD90 expression drastically changed under these conditions. However, the number of population doublings had a negative effect on the AChE activity. Cell doubling numbers and AChE activity showed an indirect correlation (Fig 4B). Together these results suggest that (i) increased proliferation, known to result in a decrease of SMDCs fusion competence [28], is responsible for lower AChE activity at the expense of differentiation, and (ii) AChE activity is independent of CD56 expression in SMDCs with increasing cell doublings. Both findings also indicate that AChE may be superior to CD56 as a potency marker as it is a true indicator of SMDCs differentiation potential.

### AChE activity testing of SMDCs can be used as a routine assay for product release

According to the ICH Topic Q2(R1) [29] quantitative tests of the active moiety in samples of drug substance or drug product or other selected component(s) in the drug product have to be validated. Relevant parameters according to EMA regulation ICH Topic Q2 (R1) as listed and described in Table 1. The potency assay we propose here showed high linearity over the specified range of 4–500 mU/mL AChE activity: coefficient of determination *r*^*2*^ = 0.9996; *r*^*2*^ = 0.9980 and *r*^*2*^ = 0.9836 for run 1, run 2 and run 3, respectively. The results are shown in Figure A in S3 Fig. The accuracy of the assay was expressed in terms of % coefficient of variance (CV). A total of 6 measurements were performed and the results are summarized in the Figure D in S3 Fig, showing less than 15%CV in every sample. The accuracy of the method was between 97 and 105%, which was found to be satisfactory. The inter-assay precision (reproducibility) of the method was performed by analysing the corresponding responses by different operators on three different days for different concentrations of standard solutions of AChE. We demonstrated the intra-assay precision of the proposed method by repeated measurement of absorbance of one SMDC batch under the same experimental conditions for minimum 20 times (Figure C in S3 Fig). The total intra-assay %CV calculated was 9.47%, which is within acceptance criteria of 20%. The intra-assay and inter-assay coefficient of variation (CV) values obtained by the proposed method were found to be lower than 20%. The values of % CV less than 20% indicate that the proposed method is reproducible for the analysis of AChE activity in SMDCs. The linear range of the assay is 4–500 mU/mL AChE activity (as defined by standard dilution range in section Material and Methods). The specificity of the assay was assessed by comparing AChE activity between non-myogenic CD56^-^ and myogenic, CD56^+^ SMDCs. Only CD56^+^ but not CD56^-^ SMDCs increase in AChE activity during differentiation, therefore demonstrating high specificity of the assay (Fig 1D and 1E). All tests conducted for validation complied with the set specifications (Figure E in S3 Fig). Only SMDCs with myogenic potential (CD56^+^ cells) have an increase in AChE activity during differentiation, therefore demonstrating high specificity of the assay.

**Table 1:** Relevant parameters for AChE potency testing of SMDCs with descriptions according to EMA Topic Q2(R1).

Relevant parameter	Description
Linearity (dilutional)	“The linearity of an analytical procedure is its ability (within a given range) to obtain test results which are directly proportional to the concentration (amount) of analyte in the sample.”
Accuracy (relative)	“The accuracy of an analytical procedure expresses the closeness of agreement between the value which is accepted either as a conventional true value or an accepted reference value and the value found. This is sometimes termed trueness.”
Precision Repeatability (intra-assay precision) Intermediate precision (reproducibility)	“The precision of an analytical procedure expresses the closeness of agreement (degree of scatter) between a series of measurements obtained from multiple sampling of the same homogeneous sample under the prescribed conditions. […]”
Range	“The range of an analytical procedure is the interval between the upper and lower concentration (amounts) of analyte in the sample (including these concentrations) for which it has been demonstrated that the analytical procedure has a suitable level of precision, accuracy and linearity.”
Specificity	“Specificity is the ability to assess unequivocally the analyte in the presence of components which may be expected to be present. Typically these might include impurities, degradants, matrix, etc. […]”

### AChE activity of differentiated SMDCs is linked to clinical efficacy of SMDCs in the treatment of fecal incontinence

We hypothesized that a link of AChE activity of SMDCs and clinical efficacy can be detected in a patient population shown to clinically improve due to SMDCs, which is defined by a significant improvement in primary efficacy variables of SMDCs treated patients compared to control treated patients. Such a patient cohort, herein termed responder population (n = 70) was identified in our phase IIb clinical trial aiming at dose finding and establishing the efficacy of SMDCs for the treatment of fecal incontinence, and concluded to clinically improve due to SMDCs [30]. This population is defined by a limited duration since onset of incontinence symptoms of ≤10 years and a high incontinence symptom severity before treatment (incontinence episode frequency, IEF) of more than 6 incontinence episodes per week [30]. Rationale for the duration selection is that prolonged incontinence duration might be associated with the time-dependent loss in muscle mass due to fibrosis within the external anal sphincter (EAS) [31]. Thus, SMDCs may not sufficiently perform their MoA of fusing with endogenous skeletal muscle fibers. Rational for the symptom severity selection is that low symptom severity patients efficiently regenerate by induction of endogenous regeneration during control treatment (needle incision, electrical stimulation). Therefore SMDCs might not be able to further improve treatment outcome in these patients. Since patients with high incontinence duration and/or low symptom severity receiving SMDCs, were found not to have a superiority in treatment outcome compared to corresponding control treatment patients, these patients were termed non-responders and concluded not to improve due to SMDCs but due to endogenous regeneration, induced via needle incision, cell carrier solution and/or placebo effects [30]. Consequently, we only expected to see a link between AChE activity of SMDCs and treatment outcome after SMDCs implantation in the responder population. Non-responders are not expected to show an AChE level-dependent effect, since SMDCs anyways are ineffective. In order to link AChE activity and clinical outcome, aliquots from every batch of SMDCs containing 2*10^5^ cells, were seeded onto gelatin coated 24-well plates and analyzed for AChE activity after 6 days of differentiation as described in Material and Methods section. Next, total AChE activity of every batch of SMDCs was determined by multiplying AChE activity per cell of *in vitro* differentiated SMDCs with the number of injected SMDCs. Both, patients of the selected responder and non-responder populations, were grouped according to the total AChE activity of the SMDCs injected intramuscularly. The groups were termed “Low”, “Medium” and “High” according to the total AChE mU_rel_ of the injected SMDCs of 1*103–2.4*10^4^, 2.5*10^4^–7.4*10^4^ and 7.5*10^4^–1.5*10^5^ mU_rel_, respectively.

**Fig 5:**
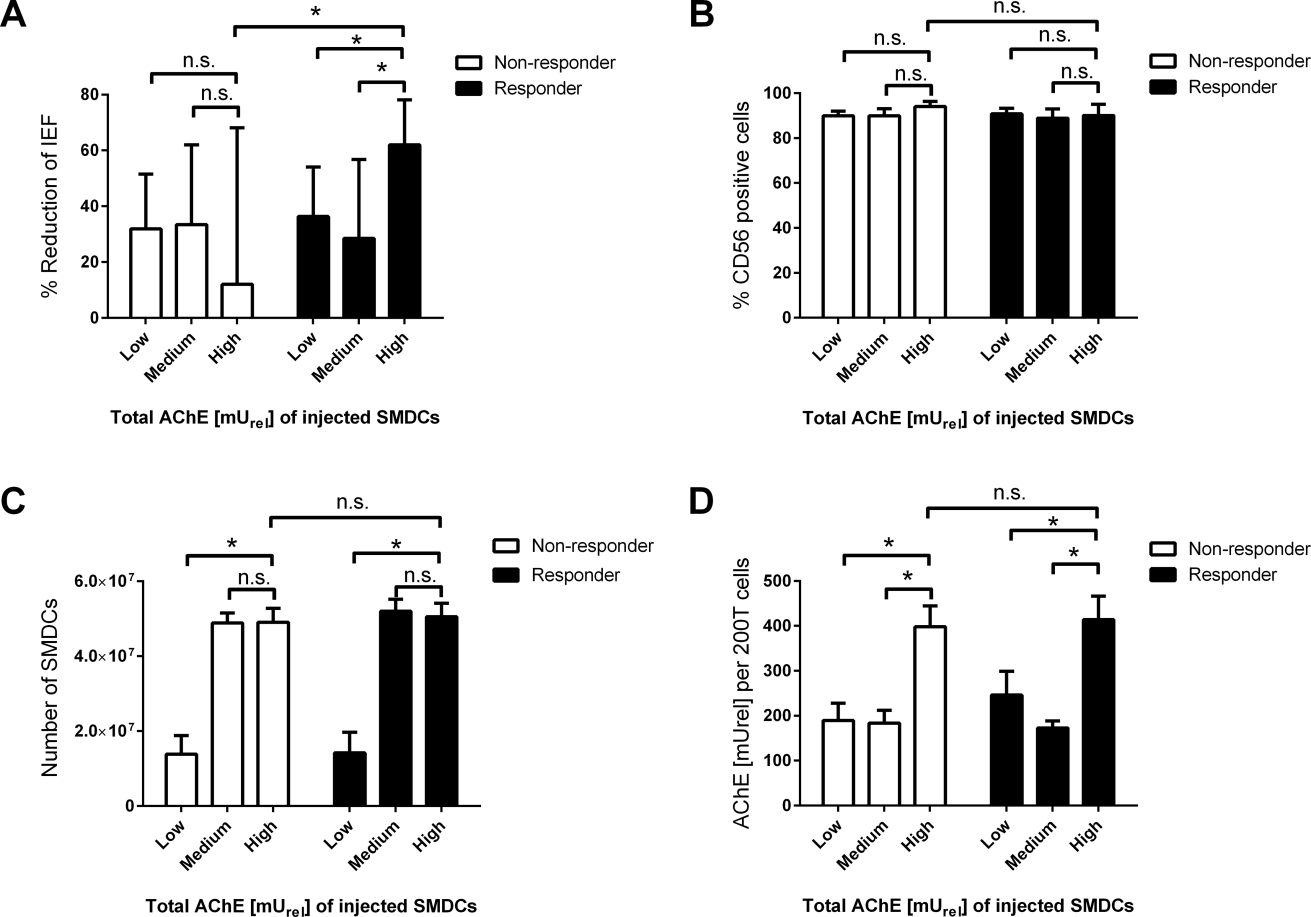
AChE activity and clinical efficacy. Percent reduction of IEF from baseline to 6 months in responders receiving Low (n = 44), Medium (n = 15) or High (n = 11) and non-responders also receiving Low (n = 48), Medium (n = 22) and High (n = 9) total AChE activity SMDC batches (A). Percentage of CD56 positive cells (B), total number of cells (C), and AChE activity [mU_rel_] per 2*10^5^ (200T) cells (D) in all SMDCs batches used for treatment of fecal incontinence in responder and non-responder patients according to total AChE activity. Data presented as mean and 95% confidence interval. Groups were compared by two-tailed unpaired *t*-test with Welch’s correction. A *p*-value below 0.05 was considered as significant (*) n.s.: not-significant.

The reduction in IEF was compared between patients injected with Low, Medium or High total AChE activity SMDCs. A reduction (mean±SD) in IEF of 38.32±56.18, 28.47±51.14 and 62.63±24.00% upon SMDCs implantation in the responder population was found for the groups with low, medium and high total AChE of injected SMDCs, respectively. A significantly higher reduction was found in high total AChE SMDCs receiving responder patients compared to low (p = 0.028) and medium (p = 0.035) total AChE SMDCs receiving responder patients, suggesting that injection of SMDCs with high total AChE activity results in better fecal incontinence treatment outcome in responders (Fig 5A).

Further, a reduction (mean±SD) in IEF of 31.87±67.77, 33.45±64.47 and 12.00±73.49% was found for the groups with Low, Medium and High total AChE of injected SMDCs in the non-responder group. No significant difference (p = 0.08) was observed between Low and High total AChE SMDCs within the non-responder group, suggesting that injection of SMDCs with high total AChE does not alter the fecal incontinence treatment outcome of non-responder patients (Fig 5).

Moreover, a significantly higher IEF reduction was found in the responder than in the non-responder population, both receiving high total AChE SMDCs (p = 0.016), suggesting that responders benefit more from receiving high total AChE SMDCs than non-responders (Fig 5A).

CD56 expression (mean±SD) of SMDCs used for injection was similar (all *p*-values >0.05) between low, medium and high total AChE SMDCs, in responders (90.83±7.95, 88.87±6.46 and 90.00±7.00% CD56^+^ cells, respectively) and non-responders (89.86±7.62, 89.94±6.99 and 94.00±3.00% CD56^+^ cells, respectively), as well as between responders and non-responders both receiving high total AChE SMDCs (Fig 5B).

Batches of low total AChE SMDCs, both in the non-responders and the responders, consisting of 1.38*10^7^±1.74*10^7^ and 1.41*10^7^±1.79*10^7^cells, respectively, contained significantly (p <0.0001) lower numbers of cells than batches within the same group with high total AChE SMDCs (non-responder: 4.91±4.85*10^6^ cells, responder: 5.05*10^7^±5.13*10^6^ cells) (Fig 5C). No significant difference in the total number of cells used for injection was found between medium and high total AChE SMDCs batches in both, non-responder (*p* = 0.93) and responder (*p* = 0.50) patients (Fig 5C). For both, non-responders and responders, AChE activity per 2*10^5^ cells was significantly higher (*p*-values <0.0001) in high AChE activity SMDCs batches (non-responder: 398±61, responder: 414±73 mU_rel_) compared to corresponding low and medium AChE activity SMDCs used for injection in non-responders (189.5±132.2 and 183.5±65.09 mU_rel,_ respectively) and responders (246±171.8 and 172.5±25.17 mU_rel_, respectively) (Fig 5D). Also no significant difference was found in CD56 expression, AChE activity per cell, and cell number injected between SMDCs injected in non-responders and responders, both receiving high total AChE SMDCs, suggesting that these parameters are not accountable for the differences in treatment outcome between responders and non-responders both receiving high total AChE SMDCs.

## Material and methods

### Isolation, cell culture and counting of SMDCs

Muscle samples of human origin were taken as a part of clinical trials (EudraCT Number: 2010-021463-32 and 2010-021871-10) and residual material of patients agreeing to further usage of their cells was used for research purpose following undersigned informed consent. The national authorities in Austria (BAGES), Germany (PEI), Czech Republic (SUKL), UK (MHRA), and Bulgaria (Bulgarian Drug Agency) (for 2010-021463-32 and 2010-021871-10) as well as Sweden (MPA) and Slovenia (JAZMP) (for 2010-021463-32) and Italy (Italian Medicines Agency) (for 2010-021871-10) authorized the clinical trials. SMDCs were isolated from muscle biopsies (*Musculus pectoralis major* or *Latissimus dorsi*) and expanded under a cGMP environment. Cells were maintained by standard cell culture methods. Briefly, cells were cultured in growth medium containing Ham’s F-10 basal medium supplemented with 10% FCS (inactivated at 57°C, 40 minutes, Life Technologies, United Kingdom), bFGF (CellGenix, Freiburg, Germany) and gentamicin (Sandoz GmbH, Austria) and incubated at 37°C, 5% CO_2_. Growth medium was changed every 2 to 3 days. For sub-cultivation and harvest, the cells were washed once with 1X PBS (Lonza Verviers Sprl, Belgium) and incubated with 1X Trypsin solution (Sigma-Aldrich Co. LLC, Germany) for 5 minutes at 37°C. Cells were rinsed with growth medium and centrifuged at 400*g for 10 minutes, supernatant was discarded and the pellet resuspended in growth medium. Counting of cells was performed on Nucleocounter™ (ChemoMetec, Allerod, Denmark) according to manufacturer’s instructions.

### Cryopreservation and recultivation

For cryopreservation of residual SMDC materials, the cell pellet (1–5 million cells) was resuspended in 1 mL cryomedium (10% DMSO, 20% FCS in Ham’s F10). The cell suspension was transferred to cryovials, frozen down to -80°C using Nalgene^**®**^ Mr Frosty (Sigma-Aldrich Co. LLC, Germany) and transferred on the next day to liquid nitrogen for storage. For recultivation of cryopreserved cells, frozen tubes were thawed in a water bath (37°C), the cell suspension was diluted with warm (37°C) growth medium and seeded in a 175 cm^*2*^ culture flask.

### Flow cytometry

Flow cytometry analysis was performed on a Guava easyCyte 6HT 2L flow cytometer (Merck Millipore, Darmstadt, Germany). Briefly, cells were harvested by trypsin at 37°C for 5 minutes, centrifuged at 400*g and resuspended in 1X PBS supplemented with 1% FCS. 4000 cells were incubated with 10 μL anti-SSEA4-PE (BD Biosciences, Pharmingen™, San Diego, USA), 5 μL IgG1-PE (Beckman Coulter Inc., France), 5 μL anti-CD56-PE (Beckman Coulter Inc., France) and 10 μL anti-CD90-PE (Beckman Coulter, Inc., France) for 15 minutes in a 1.5 mL Eppendorf tube at 4°C in dark. Cell were washed with 1 mL PBS, centrifuged at 400*g and resuspended in 200 μL of 1X PBS in a 96- well round bottom plate. After washing and resuspension, each reaction received 5 μL of viability dye 7-aminoactinomycin D (Beckman Coulter Inc., France) and plate was incubated for 10 minutes at 4°C. Cell events were acquired with Guava InCyte™ v.2.3 software. Histograms and dot-plots were generated with a minimum of 3000 events with a sample flow rate of 1.8 μL/mL. Positive staining was obtained by comparison with isotype control set as 99% negative.

### Isolation of non-myogenic cells from SMDCs

Non-myogenic (CD56^-^) cells were separated from myogenic (CD56^+^) SMDCs by Magnetic-Activated Cell Sorting (MACS). Human CD56 MicroBeads kit (MiltenyiBiotec GmbH, Bergisch Gladbach, Germany) was used for separation of CD56^+^ and CD56^-^ cells according to manufacturer’s instructions. In summary, after harvesting and counting, the cells were centrifuged at 400*g for 10 minutes, supernatant was discarded and cells were resuspended in 10 mL of MACS-buffer. After another centrifugation step (400*g for 10 minutes), the pellet was resuspended in 80 μL MACS-buffer. Subsequently, 20 μL of magnetic bead conjugated CD56 antibody was added per 1*10^7^ cells and incubated for 15 minutes at 4°C. Afterwards sorting of cells was carried out with Mini MACS Separator and non-myogenic (CD56^-^) and myogenic (CD56^+^) cells were collected in separate fractions.

### Myoblast differentiation

SMDCs were differentiated in 24-well Nunclon™ Delta Surface plastic plates (ThermoFisher Scientific, Roskilde, Denmark). Cells were seeded after coating the wells with 0.1% gelatin in 0.9% NaCl (CellGenix, Freiburg, Germany). The coating was performed by adding 300 μL of coating solution in each well and incubated overnight at 37°C. Afterwards, the coating solution was aspirated and cells were directly seeded in growth medium without any washing in a 24-well plate. Differentiation of mononucleated human myoblasts to syncytial myotubes was performed by replacing the growth medium with Skeletal Muscle Cell Differentiation medium (500 mL, PromoCell GmbH, Germany), supplemented with 10 mL of Skeletal Muscle Cell Differentation Medium Supplement Pack (PromoCell GmbH, Germany) and 240 μL gentamicin (8 mg/mL, Sandoz GmbH, Austria). Briefly, cells were seeded in a 24-well plate (120000 cells/per well) with growth medium for 1–2 days. For coating experiments, 200000 cells/well were seeded. Afterwards the growth medium was aspirated and cells were washed with Tyrode’s salt solution (Sigma-Aldrich Co. LLC, Germany). Finally, cells were covered with 1 mL of differentiation medium for 5–7 days without further medium change.

### AChE assay

AChE enyzme assay was performed according to state-of-the-art Ellman’s method with some modifications as described in the following.

#### Preparation of solutions

*American Public Health Association (APHA)* Phosphate buffer, pH 7.2 (Sigma-Aldrich Co. LLC, Germany) was prepared according to manufacturer’s instructions. In summary, 17 g of powdered mixture (monopotassium phosphate, 22.66 g/L and sodium carbonate 7.78 g/L) in 400 mL distilled water. After adding 0.5 mL Triton X-100, the mixture was dissolved on a magnetic stirrer for 30 minutes at room temperature. The final volume was made up to 500 mL in a measuring cylinder and was used without further dilution. The buffer was stored at 4°C until use. Ellman’s reagent (5,5'-*dithiobis*-*2-nitrobenzoic acid*, DTNB, 0.5 mM) was prepared freshly for each AChE assay by weighing out 2 mg in 1.5 mL Eppendorf tube. It was dissolved in 1 mL of phosphate buffer (pH 7.2 with 0.1% triton X-100) by vortexing it for 1–2 minutes. The final volume was made up to 10 mL in a 15 mL falcon tube with phosphate buffer (pH 7.2 with 0.1% triton X-100) and was stored at 4°C until use. Acetylcholine thioiodide (ATI, 5.76 mM) was prepared freshly for each AChE assay by weighing out 2 mg in 1.5 mL eppendorf tube. It was dissolved in 1.2 mL of distil water by vortexing for 1–2 minutes and then stored at 4°C until use.

#### Acetylcholinesterase (AChE) standard dilutions

AChE standard dilutions were prepared in phosphate buffer (pH 7.2 with 0.1% triton X-100) and were immediately used. A ready to use 50 U/mL AChE stock (from *Electrophorus electricus*) was purchased from AAT Bioquest^®^ Inc., Sunnyvale, CA, USA. It was diluted to prepare 1000 mU/mL of AChE according to manufacturer’s instructions, which was further diluted in a 1:2 ratio to obtain 8 different dilutions ranging from 4–500 mU/mL.

#### Colorimetric analysis

Differentiation medium was carefully removed from 24-well plate with the immediate addition of 300 μL 0.5 mM DTNB solution (prepared in phosphate buffer, pH 7.2 with 0.1% triton X-100). After 2 minutes of incubation at room temperature in dark, 50 μL of 5.76 mM ATI (prepared in distil water) was added. The reaction contents were incubated for 60 minutes at 30°C in dark followed by the OD measurement at 412 mM on an Anthos Zenyth 340rt microplate reader (Biochrom Ltd., Cambridge, UK). For standard AChE analysis, 200 μL of each dilution was mixed with 300 μL of 0.5 mM DTNB and 50 μL of 5.76 mM ATI, respectively and the OD_412_ was measured for 60 minutes in a 24- well plate.

#### Assay validation

The linearity of the method was determined by measuring AChE activity over a range of approximately 4–500 mU/mL. The linearity of the method was demonstrated by comparing AChE theoretical values *versus* measured values and was evaluated by least square regression analysis. Three runs for analytical curves of standard solutions of AChE were prepared by the dilution of the stock standard solution. All dilutions were prepared in 0.14 M phosphate buffer with 0.1% triton X-100, pH 7.2, and were tested in duplicates. A blank reaction was also included, which was composed of all reagents except AChE enzyme. Corrected OD_412_ values were obtained by subtracting blank mean measurement from mean OD_412_. Standard curve for AChE was developed in GraphPad Prism 5 software by employing ‘non-linear regression’ followed by straight-line equation. Theoretical values and measured values for AChE activity from 3 independent runs were compared over the specified range and plotted on a graph. Measured values are depicted with mean, y intercept and slope of the regression line for each run.

Since there is no accepted AChE reference method or reference standard to compare with, relative accuracy was determined by preparing standard curve as described for measurement of the linearity. Standard material was diluted to simulate four test samples with different activities within cut-off value (60% activity- theoretical activity 63–500 mU/mL). The accuracy was expressed as % recovery (% bias). Accuracy was calculated according to the formula: % accuracy = (theoretical value/ measured value) *100% (Figure B in S3 Fig). The accuracy of the method expressed as recovery (%) was between 97 and 105%.

Precision was expressed as the coefficient of variation (% CV). The mean and standard deviation (SD) of the measured absorbance is calculated to determine the %CV. The repeatability is expressed in terms of %CV. The intermediate precision of the proposed method was performed by repeated measurement of absorbance of standard solutions on 3 different days by 3 different operators. The anticipated range is 4–500 mU/mL AChE activity (as defined by standard dilutions range). In order to estimate precision of the method, one SMDC batch was seeded in three 24-well plates. After 48 hours growth medium was changed to differentiation medium and incubated for 6 days for AChE potency assay.

### Fusion index calculation

In order to determine the fusion index of SMDCs, 2*10^5^ SMDCs were seeded onto gelatin coated 24-well plates and induced to differentiate by switching to skeletal muscle differentiation medium 24 hours after seeding. After 6 days of differentiation, cells were washed twice with PBS and fixed with 4% PFA for 10 minutes. Next, cells were washed three times with PBS and stained by 2 μg/mL Hoechst33342 solution for 20 minutes. For each sample at least three fields were captured during immunofluorescence imaging and overlaid with phase-contrast images to allow easy detection of nuclei and cell boundaries. Fusion index was calculated for each captured field of vision by dividing the number of nuclei within tubes with the total number of nuclei per field following calculation of the mean for all analyzed fields. Only cells that have at least 3 nuclei were considered as myotubes. For statistical analysis at least 3 populations derived from different patients were analyzed for each group.

### Immunocytochemistry

Immunocytochemistry was performed directly in 24-well plates on differentiated myotubes. Briefly, myotubes were rinsed with PBS, fixed with 4% PFA for 10 minutes at 37°C and washed twice with PBS. Myotubes were permeabilized with 0.1% triton X-100 in PBS for 10 minutes at 37°C, rinsed twice with PBS and were proceeded further for immunocytochemistry to detect the presence of respective markers. After fixation and permeabilization of the cells, unspecific binding sites were blocked by incubation with blocking medium (0.1% Triton X-100, 3% FCS) for 1 hour at 37°C.

For fluorescent immunolabelling of SK-Myosin, cells were incubated with a mouse anti-SK-Myosin (fast) antibody diluted 1:200 in blocking medium for 90 minutes at 37°C. Afterwards, specimen were washed 3 times with PBST and subsequently stained with goat anti-mouse or donkey anti rabbit Alexa488 conjugated antibodies, diluted 1:100 in blocking medium for 1 hour at 37°C. Counterstaining of nuclei was performed by incubating the cells with Hoechst 33342 (Sigma-Aldrich Co. LLC, Germany) diluted to a finale concentration of 2 μg/mL in PBST for 20 minutes protected from light.

For enzyme-conjugated immunolabelling, cells were fixed and permeabilized with ice cold methanol for 5 minutes at room temperatures. After washing with PBS, cells were covered with Lab Vision™ Hydrogen Peroxide Block and incubated for 5 minutes at room temperature. After three additional washing steps, cells were covered with rabbit anti-desmin antibodies (Thermo Scientific, MA, USA) diluted 1:100 in PBS and incubated at 37°C for 90 minutes. Cells were washed again with PBS and covered with ready-to-use biotinylated goat anti-rabbit secondary antibodies (Thermo Scientific, MA, USA) and incubated for 60 minutes at 37°C. Afterwards, cells were washed with PBS, covered in 1:100 diluted horseradish peroxidase conjugated streptavidin (Vector, CA, USA) and incubated for 30 minutes at 37°C. Subsequently, the cells were washed and incubated with Lab Vision™ Ready-To-Use AEC Substrate System (Thermo Scientific, MA, USA) for 10 minutes. The reaction was stopped by washing with PBS and finally cells were observed by microscopy.

### Protein quantification

In order to analyze the total amount of protein within a cell population, adherent cells were first washed twice with PBS, subsequently covered with PBST (0.1% Triton X-100) and then incubated for 10 minutes at room temperature. Next, the lysate was resuspended and transferred to an Eppendorf® tube, shortly vortexed and then centrifuged for 4 minutes at 1200*g. Finally, the clear supernatant was transferred into a fresh Eppendorf tube and the protein concentration was determined using the Pierce BCA Protein Assay Kit (Thermo Scientific, MA, USA) according to the manufacturer’s instructions by measuring the OD at 540 nm with an Anthos Zenyth 340rt microplate reader (Biochrom Ltd., Cambridge, UK).

### Microscopy

Phase-contrast microscopy at 200-fold magnification was carried out both on undifferentiated and differentiated myoblasts at different time intervals in 24-well plate using Nikon Eclipse TE 2000-U microscope (Nikon Corporation, Tokyo, Japan). Syncytial myotubes containing minimum 3 nuclei per tube were photographed with the Digital Sight DS-L1 system (Nikon Corporation, Tokyo, Japan). Phase-contrast imaging at 100X was performed with Nikon Eclipse TS 100 (Nikon Corporation, Tokyo, Japan) using digital camera (DCM310) with the software ScopePhoto (v3.1; Hangzhou ScopetekOpto-Electric Co., Ltd., China). Immunofluorescence was captured directly on 24 well plate with the respective colour filter at 200X or 400X magnification with Nikon Digital Sight DS-L1 system.

### AChE data analysis

All AChE data were obtained by employing the straight-line equation developed using GraphPad Prism software (v6.0; GraphPad Software, Inc., La Jolla, CA, USA). All data were reported as mean ± SEM. Comparisons among groups were performed by *t*-test (at 95% confidence interval) using GraphPad Prism software. A *p-*value <0.05 was considered statistically significant (*).

### GeneChip microarray

Total RNA of SMDCs either cultured in growth medium or 6 days in differentiation medium was isolated by RNEasy Kit (QIAGEN, Hilden, Germany) according to the manufacturers’ instruction. Sample preparation for microarray hybridization was carried out as described in the NuGEN Ovation PicoSL WTA System V2 and NUGEN Encore Biotin Module manuals (NuGEN Technologies, Inc, San Carlos, CA, USA).Briefly, 7.5 ng of total RNA was reverse transcribed into double-stranded cDNA in a two-step process, introducing a SPIA tag sequence. Bead purified cDNA was amplified by a SPIA amplification reaction followed by an additional bead purification. 4.5 μg of SPIA cDNA was fragmented, terminally biotin-labeled and hybridized to Affymetrix PrimeView Human Gene Expression arrays for 16 hours at 45°C in a GeneChip hybridization oven 640. Hybridized arrays were washed and stained in an Affymetrix Fluidics Station FS450, and the fluorescent signals were measured with an Affymetrix GeneChip Scanner 3000 7G. Fluidics and scan functions were controlled by the Affymetrix GeneChip Command Console v4.1.3 software.

Sample processing was performed at an Affymetrix Service Provider and Core Facility, “KFB—Center of Excellence for Fluorescent Bioanalytics” (Regensburg, Germany; www.kfb-regensburg.de).

### Microarray data analysis

Summarized probe set signals in log2 scale were calculated by using the RMA algorithm with the Affymetrix GeneChip Expression Console v1.4 Software. Statistical analysis of a change in log2 scale was assessed by a ratio paired *t*-test of SMDC populations from two different patients, considering a *p*-value of <0.05 as significant (*).

### Clinical phase IIb trial

The clinical trial “Skeletal muscle-derived cell implantation for the treatment of fecal incontinence: a multicenter, randomized, double-blind, placebo-controlled, parallel-group, dose-finding clinical study” (2010-021463-32) was carried out in order to define the optimal number of SMDCs, optimal treatment population and efficacy compared to placebo for functional regeneration of the external anal sphincter. 288 female and male patients with external anal sphincter weakness or sphincter damage suffering from fecal incontinence were included in the study. 251 patients were biopsied and 244 thereof were treated with two different SMDCs doses or control (cell-free medium), by intramuscular injection of the respective substance. All SMDCs samples produced during this trial were highly enriched for CD56^+^ cells (90.01±8.81%). Patients were instructed to fill an incontinence diary for screening (day -84 to -70), before cell implantation (day -28 to -1), after implantation (day 1 to 28), prior to visit 3 (day 62 to 89), prior to visit 4 (day 152 to 179) and if patients were willing to prior to visit 5 (day 332 to 359). Diaries of day -28 to -1, day 1 to 28, day 62 to 89, day 152 to day 179, day 332 to 359 represent the data as baseline, 1 month, 3 months, 6 months and 12 months, respectively. The primary variable for efficacy was determined as the change in incontinence episode frequency (IEF) at 6 months V4 (from day 152 until day 179) compared to baseline V0 (day -28 to day -1), in each treatment group. This clinical trial was undertaken according to the ethical guidelines of the declaration of Helsinki and complies with the laws of participating countries and regulations as well as the policies and the standards defined by the international Conference on harmonization (ICH) harmonized tripartite guideline for good clinical practice (GCP) approved in January 1997 by the United States, European Union, and Japan. Patients whose data is presented in this study agreed to an analysis of their cells outside of the study to verify and improve product quality and safety (i.e. AChE activity measurement). More details on the clinical trial are available online (https://www.clinicaltrialsregister.eu/ctr-search/trial/2010-021463-32/DE, accessed on 29.11.2017).

### Linking AChE activity to clinical efficacy

An aliquot of the final formulation of SMDCs batches used for implantation into patients during the phase IIb clinical trial (2010-021463-32) was taken and induced for *in vitro* differentiation followed by AChE activity measurement as described above. In short, 2*10^5^ SMDCs were seeded onto gelatin-coated 24-well plates and differentiated in skeletal muscle differentiation medium for 6 days, followed by colorimetric analysis of AChE activity. AChE activity per cell of *in vitro* differentiated SMDCs was calculated and further multiplied with the exact total number of SMDCs used for injection (4 to 60 million) into patients. Calculated total AChE activity of the SMDCs injected into patients was used to form three groups termed “Low”, “Medium” and “High” according to the total AChE mU_rel_ of the injected SMDCs of 1*103–2.4*10^4^, 2.5*10^4^–7.4*10^4^ and 7.5*10^4^–1.5*10^5^ mU_rel_, respectively, and was compared with the percent change in IEF from baseline to 6 months post treatment. Statistical comparison between groups was performed by an unpaired *t*-test considering a *p*<0.05 as statistically significant (*), followed by Welch’s-correction due to significantly (*p*<0.05, *F*-test) different variances between groups.

## Discussion

Since the inclusion of cell- and gene- based therapies as advanced therapy medicinal products (ATMPs) into the DIRECTIVE 2001/83/EC in 2007 by the REGULATION (EC) No 1394/2007, potency must be determined for ATMPs as a part of the characterization and control strategy [32,33]. Not only in the EU but also in the US, manufacturer of cell- and gene- based therapy are requested to address the potency of their products for batch release, as stated in the regulation 21CFR610.10 [34]. Therefore, development of a potency assay for a specific ATMP has to be performed during clinical evaluation of the product. Six key points should be met by *in vitro* potency assays used for ATMPs: the potency assay must be quantitative, reflect the drug’s expected MoA and the product quality, has to correlate with the drug dose and optimally provides a link to clinical efficacy. Finally, as the potency assay is integrated into the analytical procedures for drug release, the assay has to be validated to ensure its accuracy and thus suitability for routine use. In the work presented here we established the measurement of AChE activity of *in vitro* differentiated SMDCs as a potency assay for SMDCs used in skeletal muscle regeneration, which fulfils all criteria specified above.

Autologous SMDCs were successfully used in early Phase I/II clinical trials [19,27,35] for the treatment of urinary- and fecal incontinence. Skeletal muscle is believed to regenerate by activation of quiescent satellite cells [13] that give rise to proliferating myoblasts being able to fuse with each other and existing fibers in order to restore muscle function [11–13]. The satellite cells descendants are termed myoblast and are known to express a muscle specific phenotype. Among the proteins expressed, CD56 and desmin are considered highly specific for myogenic lineage commitment and are frequently used to characterize myoblasts in culture [20,23]. The fusion of myoblasts, is considered as one of the key events necessary for myogenesis [36]. Therefore, we expect the ability of SMDCs to fuse as the MoA for functional skeletal muscle regeneration. During the fusion process of myoblasts, enzyme activities, such as AChE and creatine kinase (CK) increase [14,15,37]. AChE expressed by skeletal muscle is necessary for the functional communication between muscle and nerve, which is required for proper function of the regenerated tissue [4,17].

Increasing AChE activity was found during fusion of rabbit single nucleated myoblasts to multinucleated myotubes *in vitro* [14]. In line with the results of others [14] we could demonstrate the increase in AChE activity during human myoblast fusion using an adapted version of a previously described assay [10]. We showed that the AChE activity increase is restricted to fusion competent SMDCs and demonstrate for the first time that AChE activity directly correlates with the quantifiable fusion index of differentiated SMDCs. The AChE activity was shown in this work to be low in undifferentiated SMDCs and increase only during differentiation of fusion competent cells committed to the myogenic lineage (CD56^+^) going along with maturation demonstrated by the expression of SK-Myosin. Furthermore, we could show for the first time that increased cell doubling of a SMDC population, that is known to result in a decrease of SMDCs fusion competency [28], did result in a decrease of AChE activity during differentiation but leaving CD56 expression unchanged. These findings together support the conclusion that measuring AChE activity reflects the *in vitro* differentiation potential of SMDCs, superior to CD56, which is expressed throughout growth and differentiation of myogenic SMDCs. The superiority of AChE over CD56 as a marker for the *in vitro* differentiation potential of SMDCs, is also supported by our recent demonstration that the treatment of CD56^+^ SMDCs with TGFß1 before *in vitro* skeletal muscle differentiation results in a significant decrease in fusion competency and AChE activity without affecting CD56 expression [38].

The effective dose definition is a further pre-requisite for medicinal products and thus ATMPs (DIRECTIVE 2009/120/EC) [2]. Therefore, the putative potency parameter for cell based drugs needs to reflect the dose of the active entity (cells) within the final product formulation. Our results demonstrate that the AChE activity measured after *in vitro* differentiation of SMDCs is dependent on the number of cells induced to differentiate, thereby we conclude that AChE activity measurement is a useful parameter reflecting the cell dose. Moreover, a potency assay should provide information on the product quality, reflecting the manufacturing process of an ATMP. Thus, it should reveal possible manufacturing induced improvement or worsening of product quality in terms of its expected effectiveness. As our results suggest that CD56^-^ SMDCs do not contribute to fusion, they might be considered as a manufacturing related impurity reducing product quality. AChE activity measurement in fact correlates with the purity of SMDCs in terms of the amount of CD56^+^ cells within the population as well as their differentiation potential (fusion, maturation). Thus, AChE activity provides a link between SMDCs purity during the manufacturing and their potency in terms of *in vitro* differentiation potential.

Culturing isolated cells and supplying them with medium is vital for multiplication of cells by proliferation to reach the required dosage and is therefore part of most cell therapy manufacturing processes. However, multiple cell doublings are known to lead to myoblast senescence and thus a decrease in product quality [28]. Also it was demonstrated that myoblasts that proliferated multiple times have decreased fusion competency [28]. If AChE reflects senescence associated decrease in SMDCs quality, SMDCs which have undergone increased number of divisions *in vitro*, should have a lower potential to express active AChE during *in vitro* differentiation. As our results demonstrate that increased proliferation, known to reduce fusion competency [28], results in a decreased AChE activity, but leaving CD56 unaffected, we raise evidence that AChE activity of SMDCs reflects a manufacturing-induced decrease in product quality due to excessive population doublings, which cannot be detected by CD56 measurement. Thus we propose that AChE activity of *in vitro* differentiated SMDCs is mirroring manufacturing related changes in product quality, such as loss of differentiation potential due to excessive doubling of the SMDC population. By this, we further strengthen the evidence that AChE activity is connected to the *in vitro* differentiation potential of SMDCs and their expected MoA *in vivo*.

In order to use a potency assay as a reliable routine analytical procedure in ATMP production, proper validation is requested by regulatory authorities (ICH Topic Q2(R1)) [29]. Therefore, we pursued a theoretical approach pointing out the relevant validation parameters (linearity, accuracy, precision, range and specificity) and defined test strategies considering those parameters. The proposed measurement of AChE activity in SMDCs falls into the category of relative quantitative assays: a method, which uses calibrators (reference standards) with response-concentration calibration function to calculate the values for unknown samples. The quantification is considered relative because the reference standard is either not well characterized, not available in a pure form, or is not fully representative. As our results demonstrate, the herein described measurement of AChE activity in SMDCs is linear, accurate, precise and specific. As all tests conducted for validation complied with the set specifications, we suggest that the assay is useful for routine analysis of AChE in SMDCs in a good manufacturing practice compliant (cGMP) environment.

For use in MAA, a potency assay has to provide a link to clinical efficacy. We did expect a link between the AChE and clinical outcome to occur in patients that do respond to SMDCs treatment significantly better than to control treatment (= responder), but not in patients that do not respond to SMDCs (= non-responder) comparable to those used in the responding patients. Previously, a SMDCs responder population was found in a clinical trial aiming the treatment of fecal incontinence [30]. To test the link of AChE with clinical efficacy, patients receiving SMDCs during the mentioned clinical trial [30] were split into two populations of (1) those which did show significantly higher incontinence improvements (responders) and (2) those which did not show significantly higher incontinence improvements (non-responders) compared to their corresponding control group. Our results did show that within the responder population, patients that received high total AChE SMDCs did show better treatment outcome compared to patients that received low and medium total AChE SMDCs, suggesting a link of total AChE of injected SMDCs with clinical outcome. Low total AChE SMDCs batches contained significantly fewer cells and lower AChE activity per cell than high total AChE SMDCs batches, which suggests either AChE or the cell dose, has an effect on the treatment outcome. However, the finding that high total AChE SMDCs batches did not contain significantly higher number of cells, but significantly higher AChE activity cells and significantly higher treatment effects compared to medium total AChE activity SMDCs, clearly supports the hypothesis that AChE activity is linked to the clinical efficacy of SMDCs in the treatment of fecal incontinence patients responsive for SMDCs treatment. Together with the results that high total AChE SMDCs receiving responders show significantly higher treatment outcomes, but no difference in number of injected SMDCs, CD56 expression and AChE activity per cell, compared to high total AChE SMDCs receiving non-responders, we confirm the previously drawn conclusion that only the responder population defined by low duration since onset of incontinence and high incontinence symptom severity is responsive to SMDCs treatment [30]. Further, as all SMDCs batches used for clinical application were highly positive for CD56^+^ cells but had different treatment outcome dependent on AChE, we suggest that AChE is superior to the measurement of CD56 alone.

However, our study has several limitations. Due to the limited number of patients responding to SMDCs treatment in our phase IIb clinical trial, limited patient numbers were available for comparing AChE activity and clinical outcome. Especially, the low patient numbers in both the non-responder and responder populations receiving high AChE SMDCs, weaken the conclusions drawn. Furthermore, IEF reduction in high total AChE SMDCs receiving non-responder patients was highly variable among patients, thus limiting informative value of statistical tests. Additionally, within the responder group, medium AChE activity SMDCs receiving patients did not show superior reduction of IEF compared low AChE activity SMDCs receiving patients, which could be due to sensitivity limitations of the assay. Further, as not only AChE is known to increase during myoblast fusion, but also CK and SK-myosin, these markers might also be of interest as putative potency markers for SMDCs.

**Fig 6:**
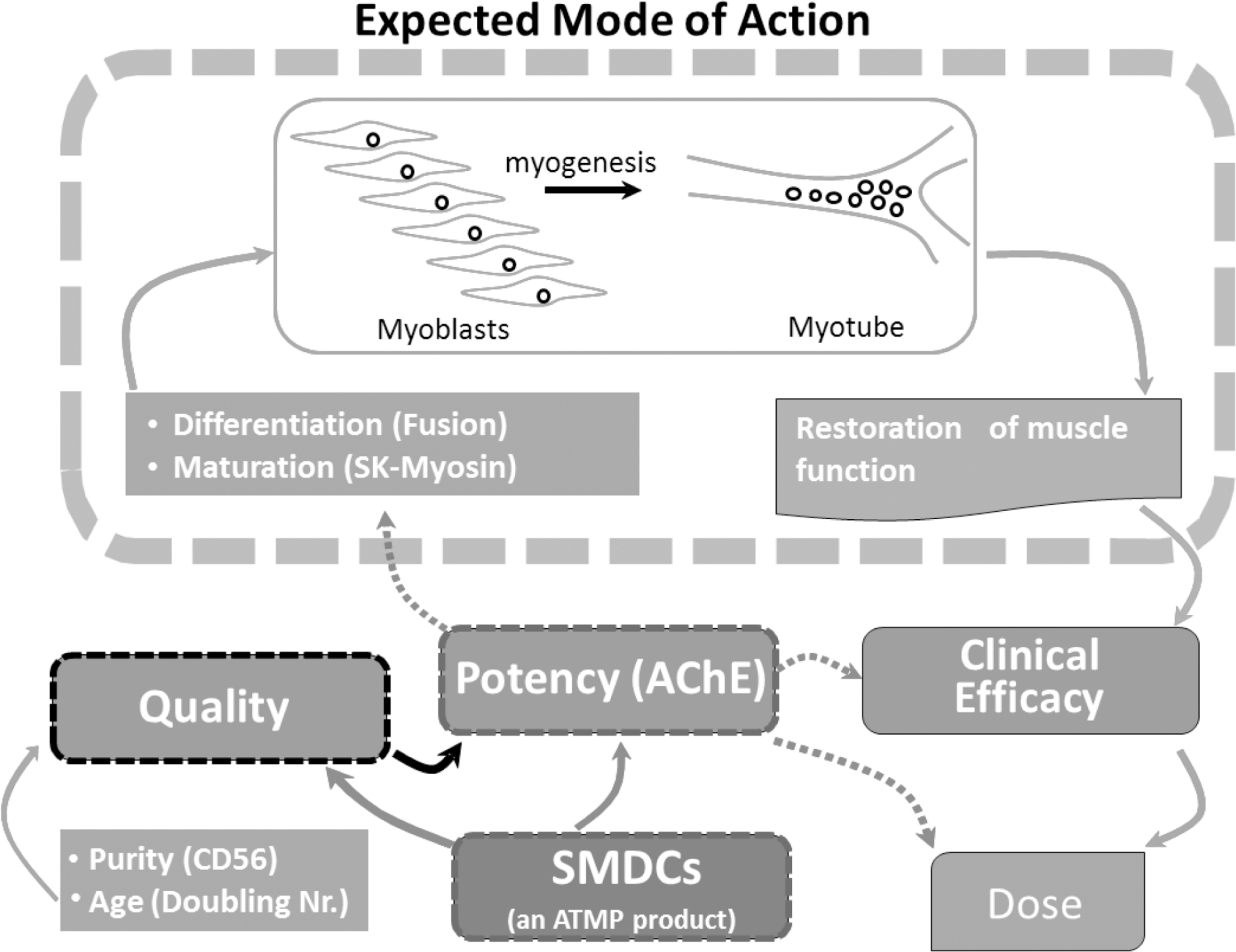
The central role of AChE as potency measure for SMDCs. SMDC quality attributes such as purity of CD56^+^ cells and population doubling numbers are connected to potency by AChE activity, which reflects the expected mode of action of SMDCs, is linked to clinical efficacy and might be useful in effective dose definition.

In summary, AChE activity was found to truly reflect the *in vitro* differentiation potential of SMDCs and to be superior to the mere use of surface markers as it reflects the amount of myogenic SMDCs in culture, their fusion competency and population doubling number, thus combining quality of SMDCs and quantification of the expected MoA. Most convincingly, our results raise evidence of a link between clinical efficacy and the AChE activity of *in vitro* differentiated SMDC preparations used in the clinic for the treatment of fecal incontinence (Fig 6). In the future, effective dose definition of SMDCs for fecal incontinence treatment might be performed by defining an AChE activity predictive for clinically significant treatment outcome.

## Supporting information

S1 Fig CD56—a myogenic lineage marker. Desmin protein expression in SMDCs with variable CD56^+^ cell percentages within the population was determined by indirect immunoreaction of 200000 SMDCs seeded on gelatin-coated 24-well plates. Scale bar = 100 μm.
(TIFF)

S2 Fig Changes in protein concentration, cell number and *ACHE* gene expression during differentiation. Protein concentration of SMDCs seeded in gelatin-coated 24-well plates was assessed before (Day 0) and 6 days after initiation of differentiation (Day 6) offer lysing cells in 200 μL 0.1% Triton X-100 (A). Cell number determined before and 6 days after initiation of differentiation by NucleoCounter® (B). *ACHE* gene expression levels before and 6 days after initiation of differentiation assessed by GeneChip microarray (Affymetrix) (C). Statistical analyses performed by two tailed ratio paired *t*-test considering a *p*-value below 0.05 as significant (*).
(TIFF)

S3 Fig Calibration curve(s) for AChE activity of standard enzyme dilutions. The assay showed high linearity over the specified range: coefficient of determination (*r*^*2*^) 0.9996; 0.9980 and 0.9836 for run 1, run 2 and run 3, respectively. The representative linear equation was y **=** 0.0032X + 0.0179 (A). Mean activity (mU/mL) for accuracy calculation (B). Method precision (% repeatability) of AChE activity (C). Intermediate precision of precision runs (OD values) (D). Summary of validation results in terms of acceptance (E).
(TIFF)
