# Supplementary figures and images for: Development of an in vitro potency assay for human skeletal muscle derived cells
Source: PLoS One. 2018 Mar 22;13(3):e0194561. doi: 10.1371/journal.pone.0194561 (PMC5864011; doi:10.1371/journal.pone.0194561)

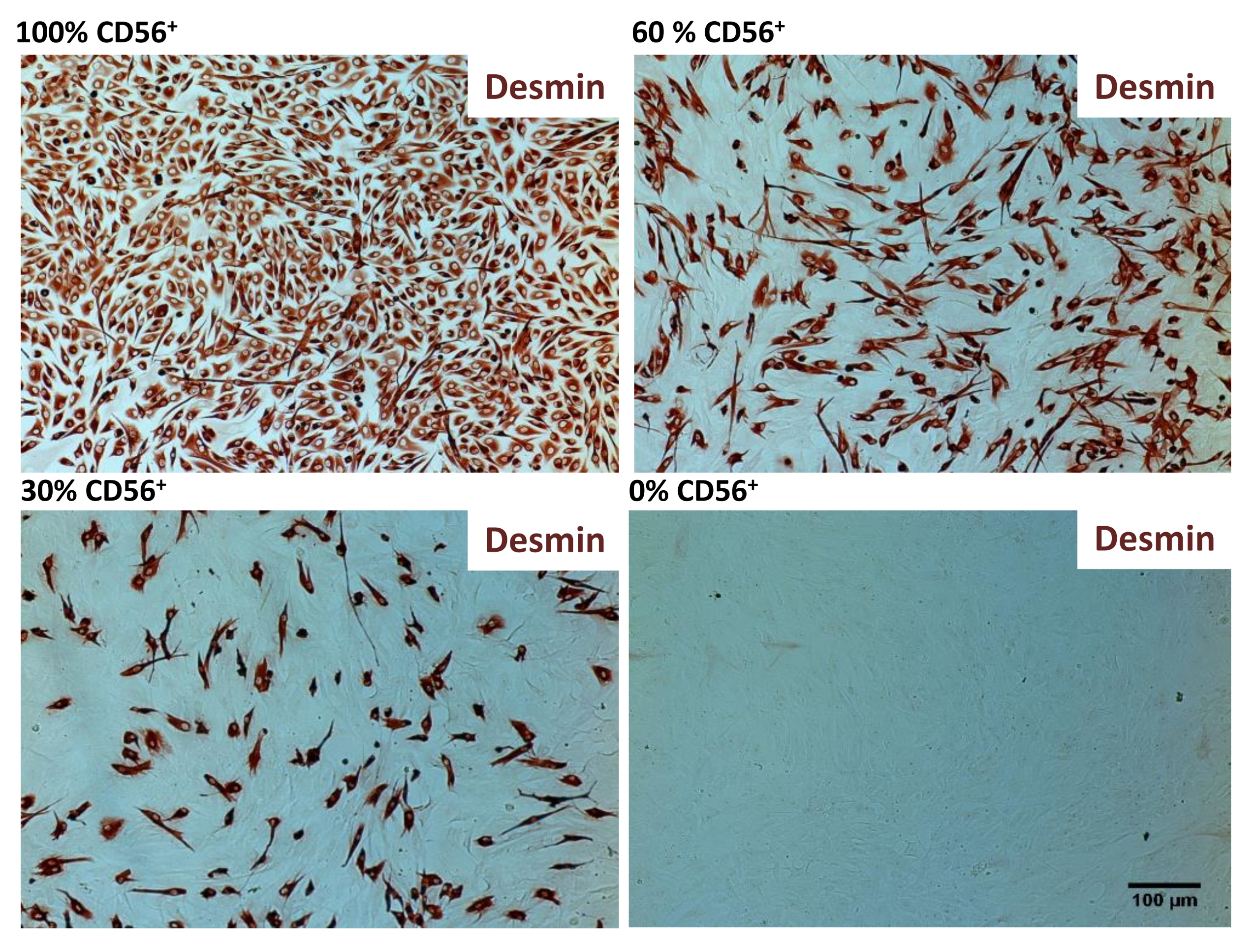

Supplement: S1 Fig — Desmin protein expression in SMDCs with variable CD56+ cell percentages within the population was determined by indirect immunoreaction of 200000 SMDCs seeded on gelatin-coated 24-well plates. Scale bar = 100 μm. (TIFF) [file pone.0194561.s001.tiff]

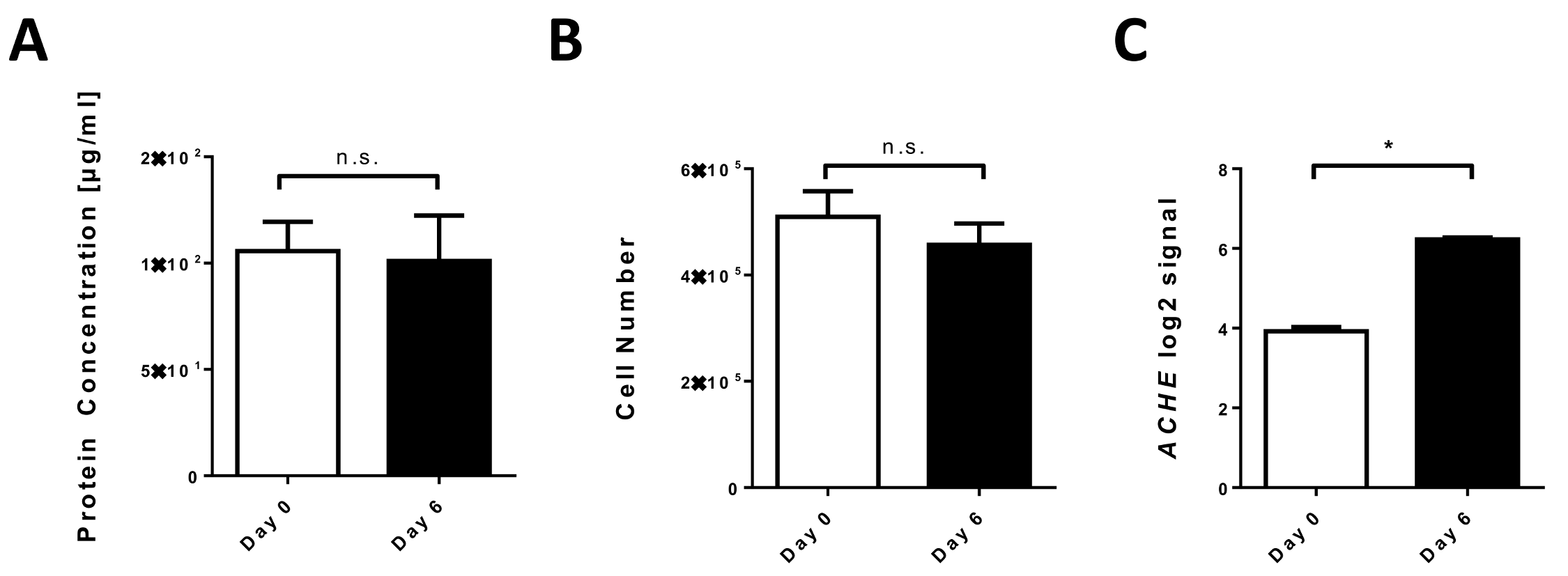

Supplement: S2 Fig — Protein concentration of SMDCs seeded in gelatin-coated 24-well plates was assessed before (Day 0) and 6 days after initiation of differentiation (Day 6) offer lysing cells in 200 μL 0.1% Triton X-100 (A). Cell number determined before and 6 days after initiation of differentiation by NucleoCounter® (B). ACHE gene expression levels before and 6 days after initiation of differentiation assessed by GeneChip microarray (Affymetrix) (C). Statistical analyses performed by two tailed ratio paired t-test considering a p-value below 0.05 as significant (*). (TIFF) [file pone.0194561.s002.tiff]

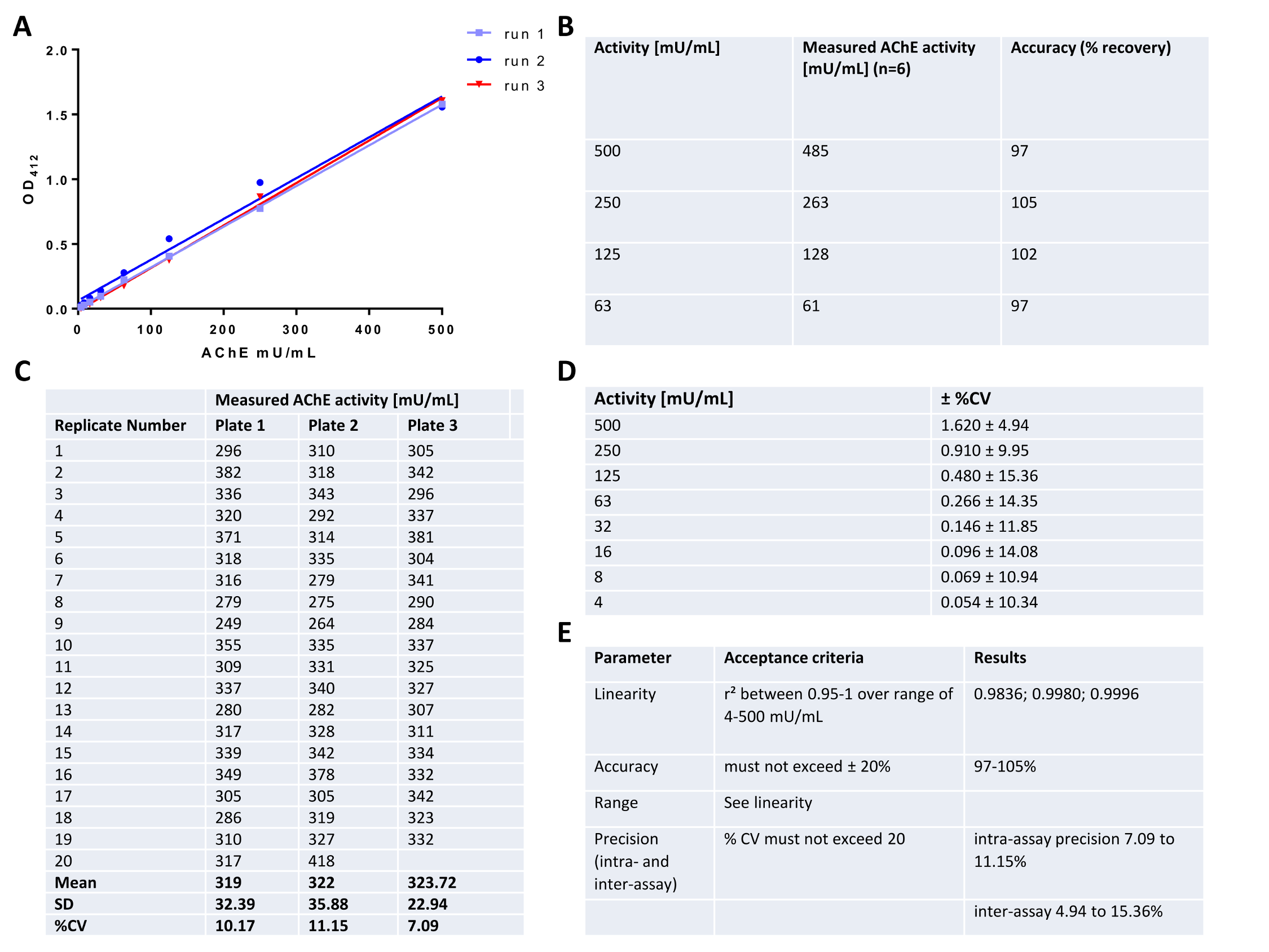

Supplement: S3 Fig — The assay showed high linearity over the specified range: coefficient of determination (r2) 0.9996; 0.9980 and 0.9836 for run 1, run 2 and run 3, respectively. The representative linear equation was y = 0.0032X + 0.0179 (A). Mean activity (mU/mL) for accuracy calculation (B). Method precision (% repeatability) of AChE activity (C). Intermediate precision of precision runs (OD values) (D). Summary of validation results in terms of acceptance (E). (TIFF) [file pone.0194561.s003.tiff]
